# From Organoids to Organ-on-Chip: Advancing Human-Relevant Models for Viral Pathogenesis and Antiviral Drug Discovery

**DOI:** 10.3390/cells15171514

**Published:** 2026-08-22

**Authors:** Vaibhav Tiwari, Joanna Choe, Aryan Vora, Ishita Kataki, Sara A. L. Roujouleh, Karin Allenspach, Michelle Swanson-Mungerson, Michael V. Volin, Sinju Sundaresan

**Affiliations:** 1 Department of Microbiology & Immunology, College of Graduate Studies, Midwestern University, Downers Grove, IL 60515, USA; mswans@midwestern.edu (M.S.-M.); mvolin@midwestern.edu (M.V.V.); 2 Department of Pharmacy, Midwestern University, Downers Grove, IL 60515, USA; joanna.choe@midwestern.edu; 3 Carl Sandburg High School, Orland Park, IL 60462, USA; avora281@gmail.com; 4 Chicago College of Osteopathic Medicine, Midwestern University, Downers Grove, IL 60515, USA; ishita.kataki@midwestern.edu; 5 College of Health Sciences, Midwestern University, Downers Grove, IL 60515, USA; sara.alroujouleh@midwestern.edu; 6 College of Veterinary Medicine, University of Georgia, Athens, GA 30602, USA; karin.allenspach@uga.edu; 7 Department of Physiology, College of Graduate Studies, Midwestern University, Downers Grove, IL 60515, USA

**Keywords:** 3D cell culture, microphysiological systems, host-pathogen interactions, preclinical drug screening, in vitro disease modeling, virology, biomedical and tissue engineering

## Abstract

**Highlights:**

**What are the main findings?**

- Organoids and organ-on-chip models provide more physiologically relevant systems for studying viral tropism, replication, dissemination, tissue injury, immune responses, and virus–host interactions than conventional 2D cultures.
- Brain, intestinal, and respiratory organoids have revealed tissue-specific mechanisms of viral infection, including HSV-1 neuroinvasion and neuroinflammation, Zika and dengue virus-induced neuronal injury, norovirus infection in intestinal models, and SARS-CoV-2 infection and pathogenesis in respiratory models.
- Incorporating immune and stromal cells, vascular components, microfluidics, and real-time monitoring enable these models to better capture the dynamic interactions between viruses, tissues, and host responses.
- The field is moving toward increasingly integrated human-relevant platforms that can model infection, inflammation, and tissue injury together, creating more predictive systems for antiviral discovery, therapeutic evaluation, and vaccine development.

**What is the implication of the main finding?**

- Organoid and organ-on-chip platforms can bridge the gap between conventional cell culture and human disease by reproducing key features of tissue organization, local host responses, and differences in viral susceptibility and treatment response. They also provide human-relevant systems for assessing drug efficacy, toxicity, and therapeutic windows.
- Advances in maturation, standardization, reproducibility, scalability, biosafety, and validation could enable these models to improve drug candidate selection, toxicity assessment, and dose optimization, making preclinical development more predictive and accelerating the development of safer, more effective antiviral therapies.

**Abstract:**

Organoid and organ-on-chip technologies are rapidly evolving platforms for viral research that integrate stem cell biology, tissue engineering, and microfluidics to recapitulate key structural, mechanical, biochemical, and cellular features of human and animal physiology. By incorporating multicellular organoids into perfused microfluidic systems, these models can provide complex, dynamic, and physiologically relevant micro-environments for investigating virus–host interactions that are difficult to capture in conventional two-dimensional cultures and static organoids. Controlled flow, shear stress, extracellular matrix organization, tissue–tissue interfaces, and multicellular signaling enable mechanistic investigation of viral infectivity, dissemination, tissue injury and immune activation. Integration of real-time imaging and biosensors further permits longitudinal monitoring of viral replication, host responses, and tissue integrity, expanding the potential of these platforms for antiviral drug discovery. Recent organoid-on-chip studies using brain, skin, vaginal, respiratory, and intestinal models have demonstrated how tissue architecture, mechanical forces, glycocalyx dynamics, and immune–stromal interactions influence viral tropism and pathogenesis. In this review, we provide a mechanistic and translational overview of organoid and organ-on-chip technologies for studying viral infections, with particular emphasis on models of herpes simplex virus (HSV)-mediated disease. We further examine advances in immune integration, multi-organ systems, biosensing, and computational approaches that are expanding the complexity and predictive potential of these models. Importantly, patient-derived organoids and organ-on-chip platforms can capture interindividual differences in viral susceptibility, host responses, and therapeutic efficacy, providing pharmaceutical research with more precise, patient-relevant data to support drug prioritization and precision antiviral medicine. Finally, we discuss key barriers to broader adoption, including organoid maturation, biological and technical variability, reproducibility, scalability, biosafety, cost, standardization, and regulatory validation. Collectively, these advances position organoid and organ-on-chip technologies as powerful human-relevant models that bridge reductionist in vitro systems and human disease, while continued optimization, standardization, and validation will be essential to realize their full potential for mechanistically informed antiviral discovery, therapeutic development, and precision medicine.

## 1. Introduction

**Figure 1:**
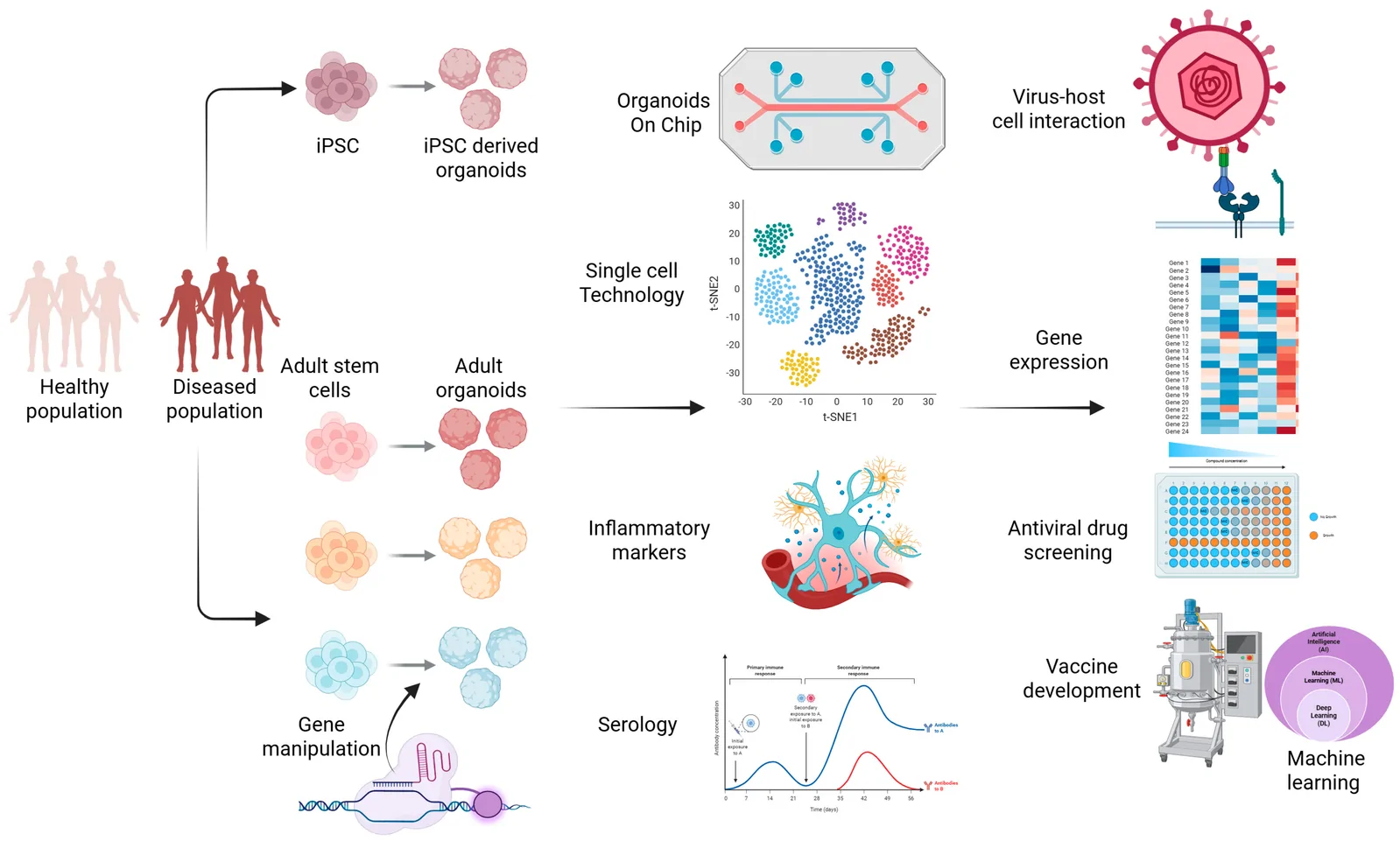
Emerging frontiers in organoid-based infectious disease research. Organoid platforms are rapidly expanding into multifunctional systems for antiviral and vaccine discovery, as well as dynamic models to dissect host–pathogen interactions. Their integration with 3D bioprinting and organ-on-chip technologies further enhances physiological relevance and scalability. In parallel, organoids support high-resolution single-cell transcriptomics, enable genome-wide association studies, and generate complex datasets for machine learning--driven predictive modeling. Collectively, these advances position organoid technology at the forefront of next-generation infectious disease research. Created in BioRender. Tiwari, V. (2026) https://app.biorender.com/citation/6964567f89adca0c9c3c2fde.

The mechanistic elucidation of virus–host interactions require experimental systems capable of reproducing the spatial, cellular, mechanical, and biochemical complexity of human tissues [1]. Viral infection is not confined to isolated molecular events within individual cells; rather, viral tropism, replication, dissemination, tissue injury, and immune responses are shaped by tissue architecture, cellular organization, extracellular matrix (ECM), glycocalyx composition, barrier integrity, mechanical forces, and interactions among epithelial, endothelial, stromal, and immune cells. Consequently, understanding viral pathogenesis and developing effective therapeutics require models that capture these interconnected biological processes within physiologically relevant tissue environments. Traditional virology has relied extensively on transformed or immortalized cell lines, including Vero and HeLa cells, because of their accessibility, scalability, and experimental reproducibility. However, immortalization can alter cellular signaling, metabolism, receptor expression, and other physiological properties, limiting the ability of these models to faithfully represent healthy human tissues [2]. Primary human-derived cell cultures provide a more biologically relevant alternative by retaining donor-specific genetic characteristics, HLA profiles, ECM interactions, and components of innate immune function [3,4,5,6,7,8,9]. Nevertheless, primary cultures are generally maintained as two-dimensional, static systems and therefore lack the three-dimensional architecture, multicellular organization, tissue polarity, vascular interfaces, and mechanical cues—including fluid shear stress and cyclic strain—that influence tissue-specific viral infection and dissemination [10]. Their finite proliferative capacity and donor-to-donor variability further complicate longitudinal studies and the investigation of persistent or latent infections. Organoid technologies have addressed several of these limitations by enabling stem and progenitor cells to self-organize into three-dimensional structures that reproduce important features of native tissues. However, conventional organoids often remain static and may lack physiological perfusion, mechanical stimulation, controlled exposure to circulating factors, and coordinated interactions with vascular, stromal, and immune compartments. Organoid-on-chip (OOCT) technology has emerged at the intersection of stem cell biology, tissue engineering, and microfluidics to address these limitations by combining the biological complexity of organoids with a more precise control of their microenvironment [11] (Figure 1). In these microphysiological systems (MPSs), preformed organoids can be incorporated into perfused chambers to preserve tissue architecture and polarity, while progenitor cells can be differentiated directly within microfluidic environments to establish spatially defined developmental and maturation gradients [12,13,14,15]. Integration of epithelial, endothelial, and stromal compartments further enables investigation of barrier function, vascular–tissue communication, immune-cell recruitment, and pathogen dissemination that cannot be adequately reproduced in conventional static cultures [13,15].

The significance of OOCT extends beyond simply increasing model complexity. By providing precise control of media flow rates, shear stress, spatial organization, chemical gradients, and multicellular interactions, these systems allow physiological variables to be manipulated and their contributions to viral pathogenesis to be directly interrogated. High-resolution imaging and microscale fluidics also provide longitudinal access to infection dynamics that are difficult to resolve in conventional cultures or animal models [16]. For example, gut-on-chip systems have demonstrated polarized release of Coxsackievirus B1 (CVB1) and inflammatory mediators from intestinal epithelium, revealing spatial features of viral infection that are difficult to capture in planar cultures [17]. Similarly, lung-on-chip models have identified macrophage colony-stimulating factor (M-CSF) as a potential biomarker of virus-triggered COPD exacerbations, highlighting the potential of these systems for clinically relevant predictive modeling [18]. These studies illustrate how OOCT can connect molecular events with tissue-level responses and identify disease mechanisms that may otherwise remain obscured. Increasingly sophisticated OOCT platforms are now being developed to reproduce tissue-specific physiological environments relevant to viral disease. A biomimetic alveolus-on-chip incorporating breathing-associated mechanical strain and an alveolar–capillary interface has enabled investigation of SARS-CoV-2 infection, inflammatory responses, and immune-cell recruitment [19]. Similarly, gut-on-chip systems have demonstrated the importance of continuous perfusion and peristaltic-like mechanical stimulation in intestinal differentiation and villus formation, while aerobic–anaerobic interfaces permit integration of the human microbiota with the intestinal epithelium to investigate complex host–microbe interactions [20]. These examples emphasize that mechanical forces, tissue interfaces, and microbial ecosystems are not merely engineering components but important biological determinants of viral infection and host responses.

**Figure 2:**
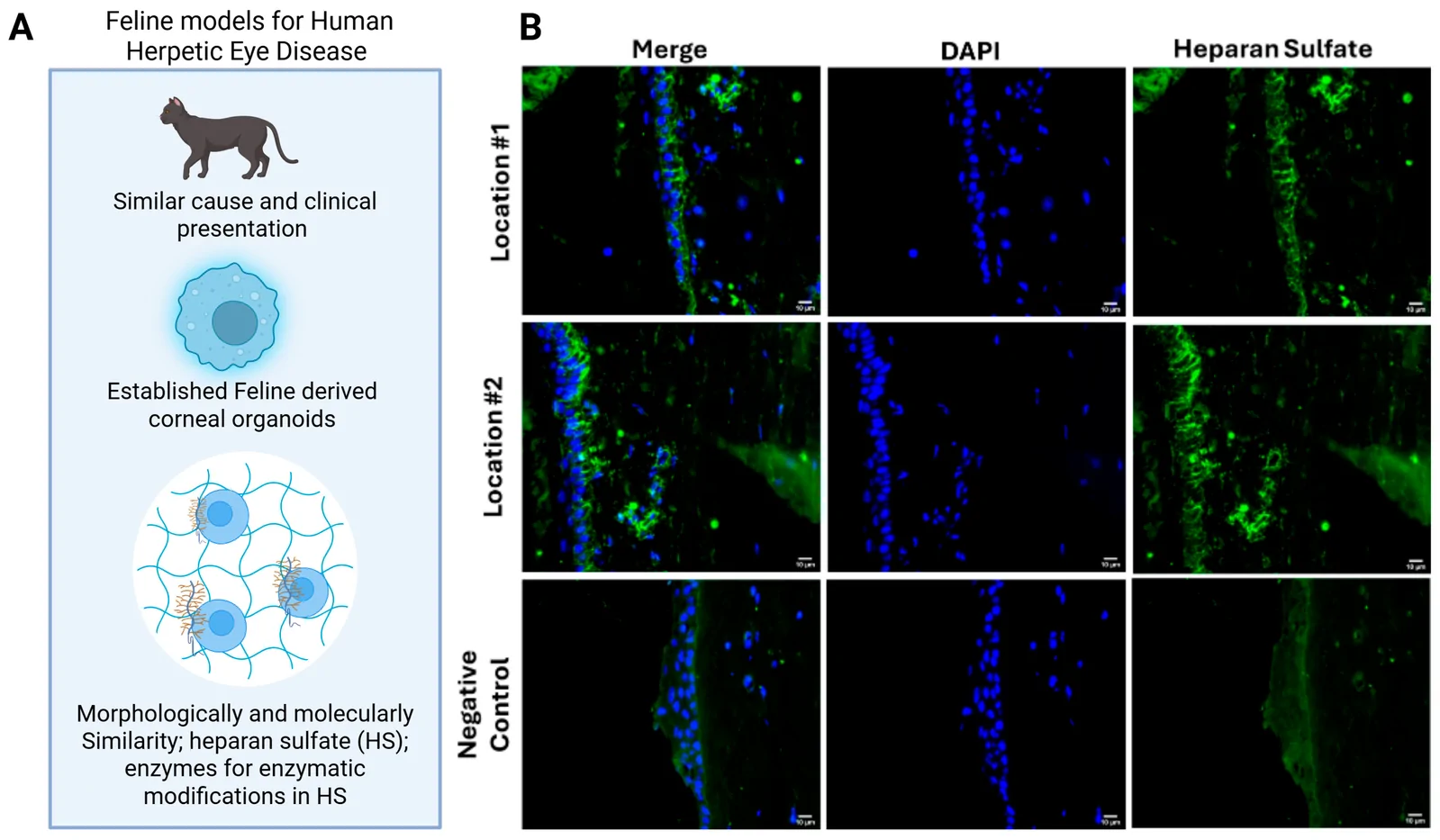
Translational relevance of OOCT: from feline models to human disease. (**A**). Feline herpesvirus is a leading cause of herpetic keratitis in cats, resulting in vision impairment. The development of standardized feline-derived corneal organoids morphologically and molecularly comparable to the human cornea provides a relevant platform to study infection dynamics. These organoids express heparan sulfate (HS) and feline 3-OST-3 enzymes, homologous to human receptors critical for HSV-1 entry. (**B**). The presence of heparan sulfate (HS) in feline corneal organoids underscores the utility of OOCT platforms for dissecting viral entry mechanisms and for developing cross-species therapeutic strategies targeting corneal inflammation for both humans and felines. Created in BioRender. Tiwari, V. (2026) https://app.biorender.com/citation/6a73b2f1b355a0df13ba70eb.

The application of OOCT is also expanding to specialized tissues and clinically important viral diseases. In ocular virology, animal-derived corneal organoid-on-chip models, including feline and canine systems, provide comparative platforms for investigating herpesvirus infection, viral keratitis, and corneal immunopathology while facilitating connections between veterinary and human disease [21] (Figure 2). OOCT systems have similarly expanded the study of sexually transmitted infections (STIs) by enabling investigation of HIV, HSV-2, and HPV within the stratified architecture of vaginal and cervical mucosa, including interactions with resident microbial communities [22,23,24]. In parallel, interconnected gut–brain and other multi-organ systems are beginning to reveal how viral persistence, systemic dissemination, and neuroimmune signaling contribute to chronic inflammation and neurological disease [25,26]. Thus, OOCT is increasingly enabling the study of both localized viral infections within specific tissues and disseminated infections involving multiple organs and physiological compartments.

A particularly important frontier is the development of immune-competent OOCT systems. Because immune responses determine viral clearance, tissue injury, inflammation, and disease outcome, models lacking relevant immune components cannot fully reproduce viral immunopathology. Next-generation platforms are therefore incorporating circulating leukocytes, tissue-resident immune populations, tissue-resident memory T cells, macrophages, and lymphoid-like structures to reconstruct dynamic immune–tissue interactions [27,28,29]. These systems enable investigation of immune-cell recruitment, activation, cytokine signaling, and resolution of virus-induced inflammation within defined tissue environments, shifting the field from static measurements of viral replication toward mechanistic analysis of virus–host–immune crosstalk [30]. This capability is particularly important for identifying therapeutic strategies that target not only viral replication but also the inflammatory and tissue-damaging pathways that contribute to disease severity.

The integration of OOCT with advanced analytical technologies is further expanding its mechanistic and translational potential. Coupling these platforms with single-cell RNA sequencing, spatial transcriptomics, proteomics, glycomics, and real-time biosensing can reveal how viral infection dynamically alters cellular states, tissue architecture, signaling networks, and glycocalyx composition [31]. Patient-derived organoids and personalized chip systems further provide opportunities to capture interindividual differences in viral susceptibility, host responses, and therapeutic efficacy. This convergence of tissue engineering, virology, immunology, and precision medicine is beginning to change how antiviral therapies can be evaluated by enabling drug responses to be examined within disease-relevant human microenvironments. Such approaches could improve compound prioritization, identify patient-specific therapeutic responses, and facilitate the development of antiviral and host-directed therapies. Collectively, OOCT is reshaping the experimental landscape of virology by providing a dynamic framework to study localized infection, systemic viral dissemination, and viral immunology within increasingly human-relevant tissue environments. At the same time, these platforms are creating new opportunities to transform antiviral drug development from largely reductionist screening toward mechanism-informed and patient-relevant therapeutic evaluation. However, important challenges—including organoid maturation, biological and technical variability, reproducibility, scalability, biosafety, cost, immune-system complexity, standardization, and regulatory validation—remain barriers to broader adoption. Addressing these limitations will be essential for translating highly sophisticated experimental models into robust, reproducible, and decision-relevant tools for antiviral development.

In this review, we examine the evolution and mechanistic applications of organoid and organ-on-chip technologies in viral research, with particular emphasis on how tissue architecture, mechanical forces, ECM and glycocalyx biology, immune integration, and multicellular interactions shape viral pathogenesis. We highlight advances across brain, ocular, skin, respiratory, intestinal, and vaginal models, and discuss emerging applications of multi-organ systems, biosensing, multi-omics, computational approaches, and patient-derived platforms. Finally, we critically evaluate the current translational barriers and future opportunities for OOCT and MPS technologies in mechanism-based antiviral discovery, host-directed therapy, and precision medicine.

## 2. Mechanistic Interrogation Across 3D Organoid and Organ-on-Chip Systems

Mechanistic interrogation of viral infection requires experimental models that integrate molecular, cellular, and tissue-level complexity under physiologically relevant conditions [1]. Three-dimensional organoid and organ-on-chip (OOCT) systems provide this integration by combining stem cell-derived organoids with microfluidic platforms that recapitulate tissue architecture, multicellular composition, dynamic perfusion, and mechanical cues [12]. These controlled and tunable microenvironments enable detailed investigation of viral attachment, entry, replication, cell-to-cell spread, tissue dissemination, and host immune responses, revealing dynamic interactions that are difficult to capture in static cultures or conventional animal models. Importantly, OOCT platforms permit longitudinal and real-time assessment of viral kinetics, host signaling, cytokine secretion, metabolic alterations, barrier integrity, and extracellular vesicle release [32,33,34,35,36]. Physiological mechanical forces represent an important component of these systems. Cyclic stretch, peristaltic-like motion, and pulsatile flow can activate mechanosensitive pathways, including YAP/TAZ, integrin–FAK, and MAPK signaling, thereby influencing receptor expression, epithelial differentiation, barrier function, and inflammatory responses [19,20,37,38,39]. Similarly, tissue–tissue interfaces—including epithelial–endothelial barriers, air–liquid interfaces, and stratified mucosal surfaces—enable investigation of intercellular communication, viral transmission, barrier disruption, and immune-mediated tissue injury [40]. By independently controlling these physical and biological variables, OOCT systems provide an experimental framework for determining how tissue context influences viral tropism and disease progression.

Beyond mechanistic studies, OOCT platforms offer increasingly sophisticated approaches for therapeutic evaluation and toxicity assessment [41]. Controlled perfusion enables more physiologically relevant drug exposure while allowing simultaneous assessment of antiviral activity, tissue toxicity, and off-target effects [15]. Functional readouts such as transepithelial electrical resistance (TEER), permeability, barrier integrity, cytokine production, and cellular viability can provide quantitative measures of epithelial and blood–brain barrier responses, supporting the evaluation of candidate therapeutics in tissue- and, increasingly, patient-relevant contexts [42,43]. The incorporation of patient-derived organoids further creates opportunities to investigate interindividual differences in viral susceptibility and therapeutic responses, supporting the development of precision antiviral interventions. The complexity of these systems can also be extended beyond individual tissues. Linked platforms modeling gut–liver, gut–brain, and other multi-organ axes enable investigation of viral dissemination, organ-to-organ communication, systemic inflammatory responses, and tissue-specific consequences of infection [44]. Where appropriate, animal-derived organoids can provide complementary comparative models for pathogens in which relevant human tissue systems remain limited, further expanding the experimental scope of organoid-based virology [44]. Together, these approaches shift infectious disease research from static endpoint measurements toward dynamic, multi-parametric interrogation of viral pathogenesis. The continued integration of bioengineering and advanced analytical technologies is further expanding the capabilities of OOCT. Bio-instructive extracellular matrices, vascularized scaffolds, three-dimensional bioprinting, and improved culture conditions are being developed to enhance organoid maturation, structural organization, longevity, and physiological function [45]. These advances are giving rise to increasingly sophisticated and functionally responsive organoid systems capable of more faithfully reproducing tissue-specific biology [46]. Integration with gene-editing technologies, single-cell transcriptomics, spatial profiling, biosensors, and machine learning further enables high-resolution analysis of virus–host interactions and identification of cellular states and molecular pathways associated with infection and therapeutic response (Figure 1).

Collectively, these advances are expanding organoid and organ-on-chip systems from experimental models of tissue biology into integrated platforms for mechanistic virology, antiviral and vaccine discovery, and precision medicine. Although challenges related to organoid maturation, standardization, reproducibility, scalability, cost, biosafety, and ethical considerations remain, continued convergence of bioengineering, microfluidics, stem cell biology, gene editing, single-cell genomics, and artificial intelligence is expected to generate increasingly functional and human-relevant models. Such platforms have the potential to accelerate the transition from mechanistic discovery to mechanism-based antiviral and anti-inflammatory therapeutics while enabling increasingly patient-centered approaches to infectious disease research and treatment.

## 3. Organoid Models for Mechanistic Virology

### 3.1. Respiratory Organoids

Respiratory viral infections remain a major global health challenge, causing substantial morbidity and mortality and placing considerable pressure on healthcare systems [47]. This threat is further amplified by zoonotic viruses that can cross species barriers, infect the human respiratory tract, and acquire adaptations that facilitate transmission and disease [48]. Developing physiologically relevant human respiratory models is therefore essential for understanding viral pathogenesis, evaluating antiviral therapies, and assessing the potential risks posed by emerging viruses [49]. The respiratory tract is a highly organized system in which the nasal and upper airway epithelium provides the first barrier to infection, while the lower respiratory tract, including the trachea, bronchi, and lungs, supports critical functions that can be severely disrupted during viral disease [50]. Viral infection is influenced by the composition and organization of epithelial cells, mucus production, ciliary activity, barrier integrity, and interactions with immune and stromal cells. Conventional cell cultures cannot fully reproduce these features, creating a need for human respiratory models that better reflect tissue structure and function. Human nasal organoids have emerged as useful models for investigating early events in respiratory viral infection. These three-dimensional systems reproduce important features of the nasal epithelium and have been used to compare the infectivity and replication of emerging SARS-CoV-2 variants [51]. Studies have demonstrated variant-specific differences in viral replication, ciliary injury, epithelial damage, and disruption of cell–cell junctions [51]. Importantly, respiratory viruses can produce distinct host responses even within similar epithelial environments. SARS-CoV-2 infection of nasal organoids has been associated with substantial ciliary and epithelial damage but relatively limited interferon-λ and mucus responses, whereas respiratory syncytial virus (RSV) induces stronger interferon-λ responses and pronounced mucus production together with ciliary injury [52]. These findings highlight how organoid models can reveal pathogen-specific differences in both tissue injury and antiviral responses. Airway and lung models provide additional opportunities to investigate viral infection at more advanced stages of respiratory disease. Differentiated human airway organoids have been used to assess the infectivity of emerging influenza viruses and to determine how viral replication is influenced by the composition and maturation of airway epithelial cells [53]. Lung-on-chip models further incorporate physiological features such as tissue interfaces and mechanical forces that are difficult to reproduce in conventional organoids [54]. Importantly, human airway-on-chip models have demonstrated that physiologically relevant tissue architecture and epithelial–endothelial interactions can substantially influence antiviral efficacy, revealing differences between drug responses observed in conventional cell-line assays and those observed in human airway tissue [55]. Respiratory organoid and organ-on-chip systems also provide opportunities for antiviral drug evaluation. Primary human airway epithelial organ-on-chip models have been used for antiviral screening against SARS-CoV-2, demonstrating the potential of these platforms to evaluate therapeutic responses within a more physiologically relevant tissue environment [56]. Access to the apical surface of airway epithelial cells is particularly important because it represents the primary site of exposure to many respiratory viruses. Conventional organoids often orient the apical surface toward the interior of the structure, which can make direct viral exposure difficult. Apical-out airway organoids overcome this limitation by positioning the apical surface toward the surrounding medium while maintaining key morphological and functional features of the human airway epithelium [57]. These models support sustained SARS-CoV-2 replication and can distinguish differences in the replicative capacity of emerging viral variants, providing a practical system for studying viral entry and replication [57]. Respiratory organoids have also expanded the study of viruses that are difficult to investigate using conventional cell cultures. Human induced pluripotent stem cell-derived respiratory organoids have been used to examine RSV-induced epithelial injury, extracellular matrix changes, and inflammatory responses [58]. Similarly, human rhinovirus C, which has historically been difficult to propagate in standard cell culture, can replicate efficiently in nasal and airway organoids, enabling mechanistic studies and evaluation of antiviral strategies [59]. These findings demonstrate that organoid models can provide suitable tissue environments for viruses whose biology is poorly represented by conventional cell lines. The increasing sophistication of airway organoid models has also created opportunities to assess the potential human infectivity of emerging influenza viruses. Long-term expandable human airway organoids can reproduce major epithelial cell populations, including ciliated, goblet, club, and basal cells [60,61]. Under appropriate differentiation conditions, these models develop abundant ciliated cells with coordinated ciliary activity and increased expression of host proteases that support productive influenza virus infection [53,62]. Importantly, differentiated airway organoids have demonstrated differences in replication between influenza viruses with established human infectivity and viruses with limited human adaptation. For example, human-infective H7N9 virus replicated efficiently, whereas H7N2 virus with limited human infectivity showed substantially lower replication [53]. Similar differences were observed between pandemic H1N1 and a swine-derived H1N1 virus [53]. These findings suggest that differentiated human airway organoids can provide a useful experimental system for evaluating the replication potential of emerging respiratory viruses and identifying viral characteristics associated with human infection.

Collectively, respiratory organoid and organ-on-chip models provide complementary systems for studying viral infection from the initial interaction with the nasal epithelium to disease processes involving the lower respiratory tract. By reproducing epithelial diversity, tissue organization, barrier function, and selected mechanical and immune features of the respiratory tract, these models enable investigation of viral replication, tissue injury, host responses, and therapeutic efficacy in human-relevant environments. Their ability to support studies of emerging viruses, difficult-to-culture pathogens, viral–bacterial co-infections, and antiviral responses further positions respiratory organoid and organ-on-chip systems as valuable tools for mechanistic virology and the development of effective interventions against current and emerging respiratory threats.

### 3.2. Gastrointestinal Organoids

The human gastrointestinal (GI) tract is a highly organized and dynamic tissue that performs essential functions in nutrient absorption, barrier protection, immune surveillance, and interactions with the resident microbiota [63]. The small intestine contains a specialized epithelial surface organized into villi and crypts and composed of multiple cell types, including absorptive enterocytes, enteroendocrine cells, tuft cells, goblet cells, and antimicrobial Paneth cells [63]. Together, these cells maintain epithelial integrity, regulate host–microbe interactions, and coordinate local immune responses. This cellular and structural complexity also creates a highly specialized environment that influences viral attachment, replication, tissue tropism, and disease severity.

Viral gastroenteritis remains a major global health problem, particularly among young children [64]. Important enteric viruses include rotavirus, norovirus, sapovirus, astrovirus, adenovirus, and enteroviruses [65]. Although extensive research has defined many aspects of intestinal biology and viral pathogenesis, understanding how these pathogens interact with the complex human intestinal epithelium has been limited by the lack of physiologically relevant experimental models. Conventional cell cultures generally fail to reproduce the cellular diversity, three-dimensional architecture, mucus barrier, epithelial renewal, and microbial interactions characteristic of the human intestine. The development of human intestinal organoids and organoid-on-chip systems has therefore provided new opportunities to investigate gastrointestinal viral infections within more representative tissue environments.

Human intestinal organoids reproduce important features of the intestinal epithelium, including multiple differentiated epithelial cell populations, tissue polarity, and crypt–villus organization. When incorporated into organ-on-chip platforms, these models can be further exposed to controlled luminal flow, mechanical forces, and physiologically relevant gradients [66,67,68]. Dynamic culture conditions can enhance epithelial differentiation, mucus production, barrier function, and other tissue characteristics that are difficult to maintain in static cultures [69]. These systems provide a controlled environment for investigating viral attachment, entry, replication, epithelial injury, and host responses while allowing specific physical and biological variables to be manipulated. They have therefore become valuable tools for studying pathogens such as human norovirus and rotavirus and for identifying host factors that regulate viral infection [67,69].

An important advantage of intestinal organoid systems is their ability to reveal how the cellular composition and physiological state of the intestinal epithelium influence viral infection. Different epithelial cell populations can support distinct stages of infection or contribute differently to antiviral and inflammatory responses. Organoid models have consequently helped identify factors associated with viral tropism, host susceptibility, and tissue injury that are difficult to resolve using conventional cell lines. Their ability to maintain epithelial barrier function and mucus production also provides an opportunity to investigate how viral infection alters the intestinal barrier and whether such changes influence secondary bacterial or microbial complications [69].

The integration of patient-derived tissues further expands the translational potential of intestinal organoids. Patient-derived organoids can retain aspects of donor-specific biology and provide experimental systems for investigating differences in viral susceptibility and responses to antiviral treatments [70]. Such models could ultimately support more individualized approaches to therapeutic development by allowing candidate treatments to be evaluated across biologically distinct patient-derived tissues. Integration with intestinal microbiota or defined microbial communities adds another important layer of complexity, enabling investigation of how microbial composition influences viral replication, epithelial responses, and disease progression [71]. This host–virus–microbiota interaction is particularly relevant because the intestinal microbiome can influence both mucosal immunity and the availability of cellular and molecular factors that affect viral infection.

Intestinal organoids also provide an increasingly useful platform for therapeutic discovery and host-directed interventions. For example, pharmacological modulation of farnesoid X receptor (FXR) signaling using compounds such as z-guggulsterone or ursodeoxycholic acid has been reported to reduce SARS-CoV-2 susceptibility in intestinal and lung organoid models, illustrating how organoid systems can identify host pathways that influence viral infection and evaluate potential therapeutic strategies [72]. Beyond antiviral screening, these models can be used to examine drug effects on epithelial integrity, inflammatory signaling, and tissue recovery, providing a broader assessment of therapeutic benefit than measurements of viral replication alone. The versatility of intestinal organoids also creates opportunities to investigate viral diseases that extend beyond the gastrointestinal tract. Because the intestine is closely connected to systemic immune system and metabolic networks, intestinal infection can influence distant tissues and contribute to extraintestinal disease. Coupling intestinal organoids with vascular compartments or other organ systems may therefore provide experimental approaches for studying viral dissemination and gut–liver or gut–brain interactions. Such interconnected models could help determine how local intestinal infection develops into systemic disease and how signals generated within the gut influence distant organs.

Despite their growing utility, intestinal organoid and organ-on-chip systems remain limited by challenges in organoid maturation, reproducibility, scalability, microbiome integration, and standardization. The complexity of the intestinal environment also creates a balance between physiological fidelity and experimental tractability. Future development will therefore require collaboration among virologists, stem cell biologists, microbiome researchers, glycobiologists, physiologists, and bioengineers to establish reproducible models that retain essential intestinal functions while remaining suitable for mechanistic studies and therapeutic evaluation.

Collectively, intestinal organoid and organ-on-chip technologies provide a powerful framework for studying enteric viral infection within a human-relevant tissue environment. By combining epithelial cellular diversity, tissue architecture, mechanical stimulation, barrier function, and microbial interactions, these models enable investigation of viral tropism, host susceptibility, immune responses, and tissue injury with a level of physiological complexity that is difficult to achieve in conventional cultures. Their integration with patient-derived tissues, microbiome models, and therapeutic screening approaches further positions intestinal organoids as valuable platforms for understanding gastrointestinal viral disease and advancing mechanism-based and patient-relevant antiviral interventions.

### 3.3. Neural Organoids

The human brain is one of the most complex organs in the body, and understanding how viral infection alters its development, structure, and function remains a major challenge [73]. The cellular diversity, regional organization, limited regenerative capacity, and specialized interactions among neurons, glial cells, and vascular components make the brain particularly difficult to reproduce in conventional cell culture models. Human stem cell-derived brain organoids have emerged as valuable experimental systems because they reproduce several features of human neurodevelopment, including tissue organization, neural progenitor populations, and aspects of regional brain architecture [74,75,76]. These models have provided new opportunities to investigate neurotropic viruses, including Zika virus (ZIKV), Japanese encephalitis virus, and severe acute respiratory syndrome coronavirus 2 (SARS-CoV-2) [77,78,79,80,81].

Early studies using two-dimensional neural cultures demonstrated that viruses can directly affect neural progenitor cells. However, many disease-associated phenotypes depend on interactions among multiple cell populations and the organization of neural tissue and therefore cannot be fully reproduced in 2D systems [82,83]. Brain organoids provide a three-dimensional environment in which viral infection can be examined within developing neural tissue. ZIKV infection, for example, preferentially targets neural progenitor cells within ventricular- and subventricular-like regions, resulting in reduced progenitor proliferation, increased cell death, and disruption of the radial glial scaffold [83,84,85,86,87]. These effects are not uniformly observed with related viruses, highlighting the importance of tissue context in determining viral tropism and disease outcome. Mechanistic studies have further identified viral and cellular pathways underlying these effects, including disruption of apical junctions by the ZIKV NS2A protein and damage to centrosome function associated with microcephaly-like phenotypes [83,87,88]. Thus, brain organoids have provided a direct connection between viral infection, disruption of tissue architecture, and developmental disease.

**Figure 3:**
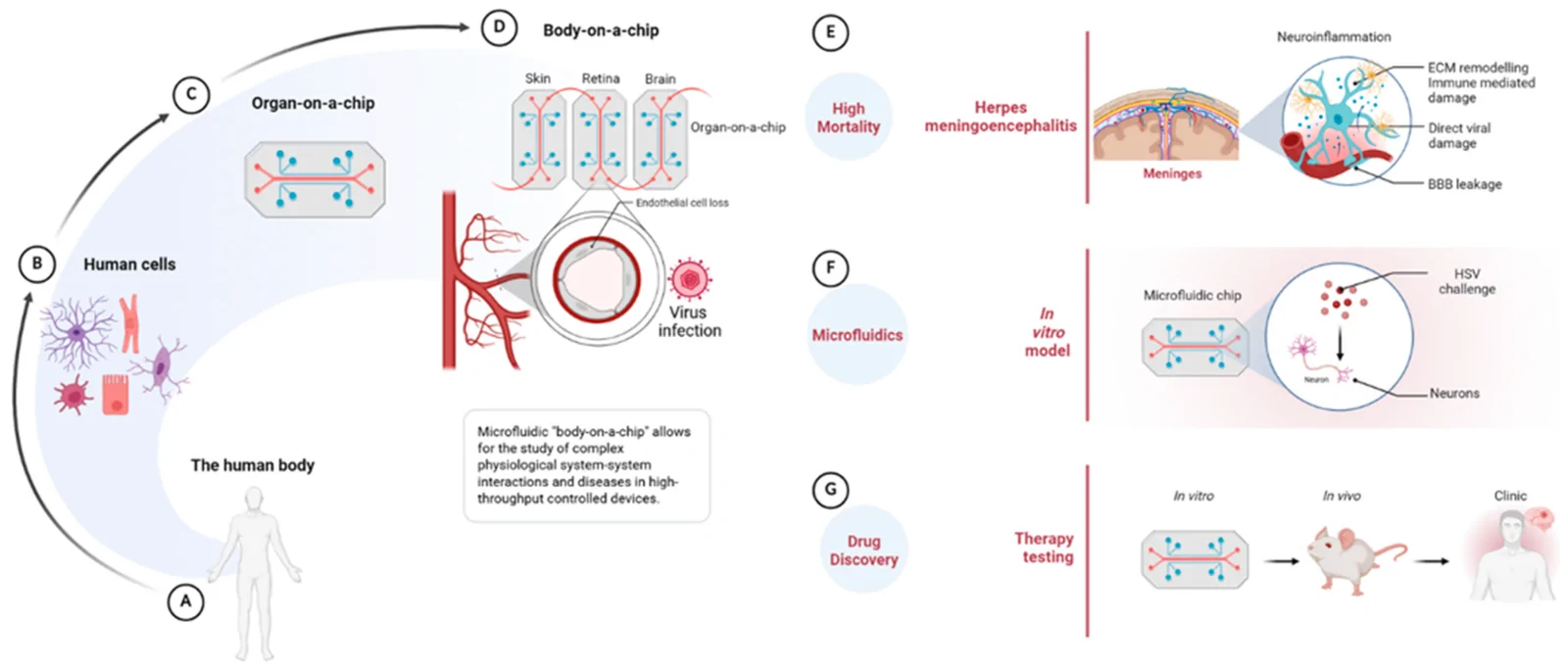
(**A**–**D**). Microfluidic organoid-on-chip platforms for human disease modeling. Organoid-on-Chip Technologies (OOCTs) integrate microfluidics with patient-derived systems to preserve native cell phenotypes, gene expression, and HLA diversity, thereby recapitulating physiologically relevant tissue microenvironments. These platforms enable high-resolution analysis of virus–host interactions, signaling dynamics, and viral evolution, while accelerating personalized antiviral and anti-inflammatory therapeutic development. (**E**–**G**). Modeling complex viral pathogenesis using OOCT. OOCT enables mechanistic modeling of herpes simplex virus (HSV)-induced meningoencephalitis by isolating susceptible neuronal subpopulations and defining molecular drivers of neuroinflammation. These systems further provide a predictive platform for antiviral and anti-inflammatory drug screening, with translational relevance to animal models and clinical studies. Created in BioRender. Tiwari, V. (2026) https://app.biorender.com/citation/6964519c89adca0c9c35bb89.

Brain organoids have also expanded our understanding of herpes simplex virus type 1 (HSV-1) infection in human neural tissue. Three-dimensional neuronal models can support both acute and latent-like infection states and have revealed profound changes in neuronal morphology, neurite organization, and cell–cell interactions following infection [88,89,90,91]. HSV-1 infection can also disrupt neuroepithelial and neuronal progenitor populations during early organoid development, suggesting that infection may interfere with developmental processes in addition to producing direct neuronal injury [89]. More recently, organoid models of herpes simplex encephalitis (HSE) have combined single-cell RNA sequencing, electrophysiological measurements, and immunostaining to characterize viral effects at multiple levels of organization [92]. These studies have revealed alterations in tissue integrity, neuronal function, and cellular transcriptional states that are difficult to resolve in conventional cultures. Importantly, these models also highlight a limitation of antiviral therapy: suppression of viral replication does not necessarily prevent virus-induced tissue injury. Although acyclovir effectively inhibits HSV-1 replication, organoid studies indicate that neuronal and neuroepithelial damage can persist despite antiviral treatment [92]. Combining antiviral therapy with agents that modulate inflammatory or cell-death pathways, including necrostatin-1 or bardoxolone methyl, has been reported to reduce tissue injury in experimental models [92]. These findings support a broader therapeutic concept in which effective treatment of viral encephalitis may require simultaneous control of viral replication and host-mediated tissue damage [93]. Brain organoids have further provided insight into the relationship between persistent viral infection and neurodegenerative disease (Figure 3). HSV-1 infection of neural organoids has been associated with amyloid-β accumulation, reactive gliosis, neuroinflammation, and neuronal loss, features relevant to mechanisms implicated in Alzheimer’s disease [91]. Antiviral treatments such as ribavirin and valacyclovir have been shown to reduce viral replication and improve some of these pathological changes [94]. Although these findings require validation in more physiologically complete models, they illustrate the ability of human neural organoids to connect viral infection with long-term changes in neuronal and glial biology. An additional strength of brain organoids is their ability to investigate intrinsic neural responses to infection. Organoid systems containing primarily neural cells allow researchers to examine cell-intrinsic antiviral pathways without the immediate influence of circulating immune cells or peripheral tissues [74]. This relative simplicity can be advantageous for identifying neuron- and glia-specific responses, although it also represents an important limitation because neuroinflammation in vivo is strongly influenced by microglia, vascular cells, and peripheral immune populations. The incorporation of microglia and other immune components into brain organoids therefore represents an important next step for modeling viral neuroimmunology more comprehensively.

Advances in organoid engineering are further increasing the physiological relevance of neural infection models. Vascularization, blood–brain barrier components, and microfluidic organ-on-chip systems can introduce controlled interactions between neural tissue, vascular compartments, and circulating factors [95]. These approaches may allow investigation of viral entry across the blood–brain barrier, vascular inflammation, immune-cell recruitment, and viral dissemination within neural tissues. At the same time, single-cell and spatial approaches provide increasingly detailed information about cell-type-specific viral susceptibility, viral tropism, and host responses [96,97]. Combining these approaches with real-time imaging and functional measurements could provide a more complete picture of how infection evolves across individual cell populations and tissue regions. The next generation of neural infection models will likely rely on integrated and hybrid systems that combine the strengths of organoids, organ-on-chip platforms, immune components, and in vivo models [98]. Transplantation of brain organoids into immunodeficient mice can promote vascularization, neuronal maturation, and gliogenesis, generating human–mouse chimeric systems with enhanced physiological complexity. In parallel, controlled co-culture of infected organoids with microglia provides an experimentally tractable approach for dissecting neuroimmune interactions. Region-specific organoids can also be combined into assembloids to reproduce interactions between distinct brain regions, while fusion of infected and uninfected organoids provides a model for examining viral spread across connected neural tissues [98].

A major advantage of human brain organoids is their ability to model aspects of human neural development that are difficult to study directly in patients. Induced pluripotent stem cell (iPSC) technology enables somatic cells to be reprogrammed into pluripotent cells while retaining the donor’s genetic background, providing a foundation for patient-specific models of viral neurological disease. Unlike conventional 2D cultures, brain organoids contain diverse neural cell populations within a three-dimensional architecture, allowing investigation of cell–cell and cell–matrix interactions that influence viral tropism, tissue injury, and host responses. Importantly, the brain is not immunologically isolated; immune surveillance involves specialized innate and adaptive immune populations within and around the CNS, including the meninges and surrounding compartments. This compartmentalized immune environment protects neural tissue while limiting excessive inflammation that could damage postmitotic neurons. Incorporating these immune components into brain organoids is therefore critical for understanding the balance between antiviral defense and inflammation during viral infection. Although brain organoids represent a major advance for modeling human neural biology, particularly when primary brain tissue is inaccessible, current systems largely reproduce features of developing rather than mature brain tissue and may exhibit variability in cellular composition and differentiation. Improved maturation, regional patterning, vascularization, and immune integration will therefore be essential to more accurately model viral neuropathogenesis and neuroimmune interactions.

### 3.4. Hepatic, Renal, and Reproductive Organoids

The study of viral infections in metabolically active organs such as the liver, kidney and reproductive tract has long been constrained by the lack of physiologically relevant human models that can recapitulate tissue architecture, multicellular complexity, and dynamic host responses [99]. The liver, a highly vascularized organ, is a primary target of hepatotropic viruses, including hepatitis viruses that cause severe diseases such as chronic liver inflammation, fibrosis, and hepatocellular carcinoma [100]. A compelling example emerges from studies on Oropouche virus (OROV), a neglected yet re-emerging arbovirus using human liver-derived organoids [101]. Clinically, patients infected with OROV often present with elevated liver enzymes, pointing toward hepatic involvement that was previously underappreciated [101]. This clinical signal finds mechanistic grounding in human liver-derived organoids, where OROV establishes productive infection, induces pronounced cytopathic effects, and triggers robust antiviral responses. Transcriptomic analyses reveal activation of interferon-stimulated genes alongside cell death pathways, highlighting a dynamic interplay between host defense and viral replication [101]. Importantly, pharmacological modulation of this axis demonstrates clear therapeutic implications: inhibition of interferon signaling enhances viral replication, whereas treatment with interferon-α suppresses infection. The antiviral drug molnupiravir, which targets viral RNA-dependent RNA polymerase, significantly reduces viral replication and tissue damage [101]. Notably, combining molnupiravir with interferon-α produces a synergistic antiviral effect, illustrating how organoid platforms can uncover combinatorial therapeutic strategies that integrate virus-targeted and host-directed interventions. This integrative power is further exemplified in studies of Hepatitis E virus (HEV), a leading cause of acute viral hepatitis worldwide [102]. Using induced pluripotent stem cell (iPSC)-derived multilineage organoids spanning the liver, intestine, and brain, researchers have demonstrated that HEV exhibits remarkable pan-tissue tropism. In liver organoids, infection spans hepatocytes, cholangiocytes, macrophages, and stellate cells, leading to elevated interleukin-6 levels, reduced albumin and Factor IX secretion, and increased aminotransferases hallmarks of hepatic injury [103]. In intestinal organoids, HEV disrupts epithelial barrier integrity, downregulates tight junction proteins, and induces epithelial–mesenchymal transition alongside proinflammatory signaling [103]. Strikingly, brain organoids reveal neuronal tropism, with infection of glutamatergic, dopaminergic, and GABAergic neurons, as well as glial populations, accompanied by alterations in neuronal composition [103]. Treatment with ribavirin partially rescues these phenotypes across organ systems. Together, these findings establish a unified, cross-organ model of HEV pathogenesis, demonstrating how organoid systems can reveal systemic dimensions of viral disease that remain inaccessible in conventional models. Similarly, Hepatitis B virus (HBV) research has been transformed by the development of hiPSC-derived hepatic organoids that faithfully support the complete viral life cycle [104]. These systems maintain stable expression of the sodium taurocholate cotransporting polypeptide (NTCP), enabling authentic viral entry, followed by replication, covalently closed circular DNA (cccDNA) formation, antigen secretion, and production of infectious progeny [104]. Beyond viral replication, these organoids recapitulate key features of chronic HBV infection, including suppression of hepatocyte functional genes such as ALB and CYP3A4, alongside induction of fibrosis-associated markers like COL1A1, reflecting early extracellular matrix remodeling [104]. Critically, this platform enables mechanistic dissection of antiviral therapies: tenofovir reduces viral DNA and antigen production without affecting cccDNA, whereas bulevirtide specifically blocks viral entry. At the same time, drug-induced hepatotoxicity can be assessed within the same system, highlighting the dual capacity of organoids for efficacy and safety evaluation. This positions hepatic organoids as a scalable and predictive platform bridging basic virology and translational drug development. In case of hepatitis C virus (HCV) infection, which is now readily treatable with antiviral therapies, however, it remains associated with metabolic complications such as nonalcoholic fatty liver disease (NAFLD) [105]. Progress in understanding the mechanistic link between HCV and NAFLD has been limited by the lack of physiologically relevant in vitro models. A recent study developed a novel multicellular liver organoid system derived from human pluripotent stem cells, incorporating hepatocytes, hepatic stellate cells, and macrophages that differentiate into Kupffer-like cells [105]. This co-culture model recapitulates key aspects of liver microenvironment and intercellular communication. Using this platform, authors demonstrate that HCV infection enhances lipid accumulation through upregulation of host lipogenesis, an effect amplified by the presence of Kupffer-like cells [105]. Conversely, fatty acid treatment promotes viral replication, inflammation, and fibrotic responses [105]. Importantly, the model reliably mirrors clinical responses to advanced nonalcoholic steatohepatitis (NASH) therapeutics, underscoring its utility as a physiologically relevant system to study HCV-associated nonalcoholic fatty liver disease (NAFLD) progression. Similarly, dengue virus directly targets hepatocytes, and human stem cell-derived liver organoids have been shown to be highly susceptible to DENV-2 infection, with preferential infection of proliferating hepatocyte-like cells leading to mitochondrial damage, cell death, and severe dengue-like tissue pathology [106]. Importantly, oxyresveratrol and omaveloxolone exhibit potent antiviral effects by activating the NRF2 pathway, thereby reducing oxidative stress and preserving mitochondrial function, highlighting a promising host-targeted therapeutic strategy [106]. Organ-on-a-chip and organoid-on-chip platforms have expanded the study of viral infections across multiple organ systems by recreating vascularized, perfused, and physiologically relevant tissue microenvironments. Kidney-on-chip models also enable controlled viral inoculation and detailed analysis of host–pathogen interactions [32]. In particular, a distal tubule-on-a-chip model of pseudorabies virus infection demonstrated that viral exposure disrupts sodium transporter function, damages epithelial barrier integrity, and alters microvilli structure, leading to impaired sodium reabsorption and consequent electrolyte imbalance [32]. Together, these findings highlight the strength of organ-on-a-chip systems in reproducing organ-level functions and uncovering mechanisms of virus-induced dysfunction. Inter-organ communication is also being captured with increasing sophistication. Gut–liver axis models demonstrate how viruses such as hepatitis E virus can transit between tissues, with bile acids acting as critical regulators of viral replication through pathways such as farnesoid X receptor (FXR) signaling [107]. These findings underscore the importance of systemic context in viral infections, where metabolic and signaling networks across organs shape disease progression. Beyond internal organs, organoid-on-chip platforms are also illuminating host–microbiome–virus interactions at mucosal surfaces. Human vagina-on-chip systems, for instance, reveal how Lactobacillus-dominated microbial communities maintain epithelial integrity, low pH, and anti-inflammatory states, whereas dysbiotic communities dominated by Gardnerella vaginalis disrupt barrier function and elevate inflammation [24]. These models provide a powerful framework to study how microbial ecology could modulate medically significant viral (HSV, HPV and HIV) transmission and pathogenesis in a tissue-specific context. Similarly, a recent study developed a human cervix organ-on-a-chip model to better understand cervico-vaginal microbiome dynamics and bacterial vaginosis (BV) [108], a prevalent condition strongly associated with adverse reproductive outcomes. Existing in vitro and animal models fail to replicate the complex mucus-rich, hormonally responsive cervical microenvironment, limiting mechanistic insight into host–microbiome interactions. The newly developed Cervix Chip, incorporating primary cervical epithelial cells and stromal fibroblasts under dynamic flow, successfully reproduces key features of endo- and ectocervical physiology, including mucus production, barrier function, and hormonal responsiveness [108]. Importantly, it enables stable, long-term co-culture with microbiota and more faithfully models in vivo-like host–microbe interactions, providing a powerful platform to study BV pathogenesis and test therapeutic strategies. In summary, hepatic, renal, and reproductive multicellular organoid and organ-on-chip models are expanding viral research from measurements of viral replication toward a more integrated understanding of tissue-specific disease including chronic hepatitis B virus infection and hepatocellular carcinoma. Their ability to reproduce specialized cellular functions, barrier properties, metabolic signals, and host–microbiome interactions provides a direct link between viral infection and organ dysfunction. Importantly, these models can also resolve human-specific mechanisms of viral host tropism and evaluate how clinically relevant medications influence infection. For example, human iPSC-derived kidney organoids with improved proximal tubule maturity have been used to define SARS-CoV-2 entry into renal cells, identifying ACE2 as the primary receptor and demonstrating that the commonly prescribed ACE inhibitor lisinopril has minimal effects on renal infection. Such findings illustrate the value of human organoid models for addressing clinically relevant questions that are difficult to resolve in conventional systems. More broadly, organoid and organ-on-chip technologies are evolving beyond models of viral infection into integrative platforms that connect clinical observations with mechanistic biology across tissues and biological scales [22,23,24]. By enabling simultaneous investigation of viral replication, host responses, tissue injury, organ dysfunction, and therapeutic responses within human-relevant systems, these platforms are reshaping the study of viral diseases and their associated complications. As interconnected and patient-derived models continue to develop, they offer an increasingly powerful approach for understanding localized and disseminated viral disease and evaluating antiviral and host-directed therapies within specific human tissue environments.

## 4. Cross-Species Modeling of Herpesvirus Corneal Infection Using Feline Organoids

Feline corneal organoids provide a valuable bridge between experimental virology and naturally occurring ocular herpesvirus disease. Derived from adult limbal stem cells, these three-dimensional models reproduce important features of the native cornea, including a stratified epithelium, progenitor cell populations, epithelial polarity, and barrier-forming junctions [21] (Figure 2). They also preserve interactions between epithelial and stromal cells that contribute to tissue repair, barrier maintenance, and inflammatory responses. These features provide a more representative environment for studying corneal infection than conventional monolayer cultures and make feline organoids useful for both veterinary and comparative ocular research [109]. The relevance of this model is particularly evident for feline herpesvirus-1 (FHV-1), a naturally occurring alphaherpesvirus that causes ocular disease in cats and shares important pathological features with HSV-1 infection of the human cornea [109]. Both viruses infect the corneal epithelium and can cause epithelial destruction, ulceration, stromal inflammation, neovascularization, scarring, and vision loss, particularly following recurrent infection. Feline corneal organoids therefore provide an opportunity to examine herpesvirus infection within a tissue environment that closely reflects the anatomical site of disease while avoiding some of the limitations of conventional animal models.

An important strength of these organoids is their ability to preserve the native corneal architecture and molecular determinants of herpesvirus infection [110]. The composition and sulfation of heparan sulfate proteoglycans influence viral attachment, while viral glycoproteins such as gB, gC, and gD mediate key steps in entry and cell-to-cell spread. The presence of glycan-modifying enzymes, including 3-O-sulfotransferases, further provides an opportunity to investigate how tissue-specific glycan structures influence viral tropism and susceptibility [111]. Rather than studying these interactions in isolation, feline organoids allow glycan–virus interactions to be examined within a structured epithelial environment. These models also provide an important system for investigating the relationship between viral replication and corneal inflammation. Infection can be examined alongside innate antiviral pathways, including type I interferon and NF-κB signaling, allowing researchers to distinguish direct viral effects from host responses that contribute to tissue injury. This distinction is particularly important in herpetic eye disease, where clinical damage may persist even after viral replication is controlled. Understanding how viral infection, epithelial injury, and inflammatory signaling interact may therefore reveal therapeutic targets beyond viral replication alone. The translational potential of feline corneal organoids extends to therapeutic development. They can be used to evaluate antiviral compounds, inhibitors of glycan-dependent viral entry, and host-directed therapies designed to limit inflammation, tissue damage, and impaired repair [112,113]. Because the model also retains epithelial progenitor populations, it provides an opportunity to investigate tissue recovery following infection and to evaluate strategies aimed at restoring corneal epithelial integrity [114]. This combination of infection, inflammation, and tissue repair makes the model particularly relevant to recurrent or severe herpetic keratitis.

Despite these advantages, feline corneal organoids do not fully reproduce the complexity of the intact cornea. Donor-to-donor variability, incomplete representation of vascular and immune components, and species-specific differences in ocular biology remain important limitations. Incorporating immune cells, vascular elements, and patient-relevant molecular profiling could further improve their physiological and predictive value. An important next step would be the development and use of healthy, adult stem cell-derived feline corneal organoids as an experimentally tractable model for herpesvirus infection. These organoids could be infected in vitro to recapitulate key features of herpesvirus-induced corneal disease while preserving the cellular organization and tissue-specific characteristics of the feline cornea. Such models would provide an opportunity to investigate herpesvirus-induced changes across corneal cell types, identify host and viral determinants of disease, and evaluate therapeutic strategies in a genetically and physiologically relevant tissue context. Overall, feline corneal organoids provide a distinctive comparative platform in which herpesvirus entry, glycan-dependent tropism, epithelial injury, inflammation, and tissue repair can be examined within a single experimental system. By connecting naturally occurring feline herpesvirus disease with mechanisms relevant to human HSV-1 ocular infection, these models may facilitate the identification of conserved viral and host targets and accelerate the development of therapies that address not only viral replication but also the inflammatory and tissue-destructive processes that ultimately drive corneal disease and vision loss.

## 5. Modulation of Heparanome and Extracellular Matrix by 3D Organoid-on-Chip Systems Bottom of Form

The extracellular matrix (ECM) is a dynamic component of human tissues that provides structural support while actively regulating cell adhesion, polarity, differentiation, signaling, and tissue repair [115,116]. Within this environment, the heparanome, which includes heparan sulfate (HS), heparan sulfate proteoglycans (HSPGs), and the enzymes that synthesize and modify HS, plays an important role in regulating cell–cell and cell–matrix communication [117]. Because HS binds growth factors, cytokines, chemokines, and viral proteins, changes in its abundance, sulfation, or distribution can influence both tissue function and viral susceptibility. Viral infection can substantially alter the heparanome and surrounding ECM. Infection can change the expression of heparan sulfate (HS) and HS-modifying enzymes, including NDSTs, EXT1/2, HS2ST, HS3ST, and HS6ST, thereby altering sulfation patterns that influence viral attachment and entry [118]. HSPGs such as Syndecans and glypicans may also be upregulated, redistributed, or shed during infection, affecting viral spread and cellular signaling [119]. In addition, activation of heparanase can promote HS degradation and ECM remodeling, potentially facilitate viral dissemination, and contribute to inflammation, tissue injury, fibrosis, and abnormal vascular responses [118,120]. Thus, the heparanome is a dynamic component of the tissue microenvironment that influences both viral infection and host responses resulting in cell and tissue injury. The ECM used to generate organoids is an important determinant of their structure and function. Common matrices such as Matrigel provide a complex mixture of ECM proteins and growth-promoting factors that support organoid formation and differentiation. However, their poorly defined composition and batch-to-batch variability can reduce reproducibility and make it difficult to identify which matrix signals regulate specific cellular responses. Decellularized ECM (dECM) hydrogels retain tissue-specific biochemical signals and can more closely reproduce the native tissue environment, although variation in tissue source and processing remains a challenge. Synthetic and engineered hydrogels offer greater control over matrix composition, stiffness, degradation, and ligand presentation, allowing individual ECM properties to be systematically examined. However, highly defined synthetic matrices may not fully reproduce the complex biochemical environment of native tissues. Selecting or engineering an appropriate ECM is therefore essential for developing organoid models that accurately reproduce tissue-specific structure and function. Three-dimensional organization further influences the relationship between cells, ECM, and the glycocalyx. Unlike planar cultures, organoids establish tissue polarity and spatially organized cell–matrix interactions. HSPGs are distributed according to cellular polarity, with Syndecans commonly enriched at basolateral surfaces and glypicans associated primarily with apical or luminal membranes [121,122]. This organization creates distinct HS-rich microenvironments that regulate growth-factor signaling, barrier function, tissue development, and viral access to host cells. ECM composition can also influence the expression and activity of HS biosynthetic and modifying enzymes, establishing a dynamic relationship between matrix composition, glycocalyx organization, and cellular signaling [121,123].

Organoid-on-chip systems further improve the physiological relevance of these models by introducing controlled fluid flow and mechanical forces. Perfusion, shear stress, and tissue deformation can influence glycocalyx organization, HS turnover, ECM remodeling, and cellular signaling [124]. These forces also regulate the distribution of nutrients, growth factors, and signaling molecules, generating spatial and temporal gradients that are difficult to reproduce in static cultures. The relationship is bidirectional: the ECM and mechanical environment influence HS synthesis and organization, while changes in HS can alter cell adhesion, signaling, and tissue behavior. This dynamic microenvironment is particularly important for understanding viral infection. Many viruses, including herpesviruses and SARS-CoV-2, use HS as an attachment or entry factor [118]. However, HS composition, sulfation, and accessibility differ among tissues and may change during infection. Organoid and organoid-on-chip models can therefore help determine how tissue-specific ECM and HS characteristics influence viral tropism, attachment, entry, cell-to-cell spread, and dissemination. These models can also reveal whether changes in the glycocalyx are directly induced by viral infection or occur as part of the host response to tissue injury and inflammation. The importance of the ECM–heparanome axis is particularly evident in ocular disease. In corneal and retinal models, ECM components such as collagens, laminins, fibronectin, and proteoglycans provide structural and biochemical signals that regulate epithelial organization, cell migration, differentiation, and tissue repair. Following injury or viral infection, remodeling of these components can determine whether tissue returns to normal function or progresses toward persistent inflammation, fibrosis, and scarring. Ocular organoid and organoid-on-chip systems therefore provide an opportunity to investigate how viral infection alters ECM and glycocalyx organization and how these changes contribute to impaired wound healing and vision-threatening tissue damage. Decellularized and engineered ECM scaffolds may additionally provide controlled environments for evaluating regenerative therapies, antiviral compounds, and host-directed treatments designed to restore tissue integrity.

Recent advances in glycomics, mass spectrometry, imaging, and single-cell analysis now enable increasingly detailed characterization of HS structure and distribution [125]. Combining these approaches with organoid-on-chip systems could reveal how specific sulfation patterns, HSPGs, and HS-modifying enzymes change during infection and how these changes influence viral and host signaling. CRISPR-based manipulation of HS biosynthetic and modifying enzymes could further establish causal relationships between individual components of the heparanome and viral susceptibility. Similarly, tunable ECM systems could be used to determine how matrix composition and mechanical properties influence glycocalyx organization, viral infection, inflammation, and tissue repair. Overall, integrating ECM biology, glycobiology, and organoid-on-chip technology provides a powerful framework for studying the dynamic ECM–heparanome–virus interface. Rather than focusing solely on viral replication, these models allow simultaneous investigation of tissue architecture, matrix composition, glycocalyx organization, inflammation, and repair. Such integrated systems may improve the physiological relevance of human infection models and support the development of novel antiviral therapies that address both viral infection and the tissue damage responsible for chronic disease.

## 6. Cellular Architecture and Organoid-on-Chip Models for Mechanistic Dissection of Viral Pathogenesis

Viruses depend on host cellular machinery to enter cells, move within them, replicate, and spread to neighboring cells. Cytoskeletal networks, particularly actin filaments and microtubules, are central to these processes and can also influence viral evasion of host defenses [9,126,127]. Although conventional two-dimensional (2D) cultures have been valuable for defining these mechanisms, they do not reproduce the three-dimensional organization, cellular diversity, polarity, or tissue-specific microenvironment of human tissues. Organoids provide a more physiologically relevant setting by organizing multiple cell types within a defined three-dimensional architecture and preserving cell–cell and cell–matrix interactions that influence viral infection [15]. This allows viral entry, intracellular trafficking, cell-to-cell spread, and tissue injury to be examined within a tissue-like environment rather than as isolated cellular events. Advanced imaging has further increased the mechanistic value of organoid models. Confocal and lattice light-sheet microscopy can visualize viral movement through actin- and microtubule-dependent pathways and follow changes in cell morphology during infection [128]. Three-dimensional mammary spheroid models, for example, retain cellular structures such as filopodia and tunneling nanotubes that may contribute to intercellular viral transfer [129,130]. Their organization within a multicellular tissue environment provides an opportunity to determine how these structures contribute to viral dissemination. Importantly, cytoskeletal behavior is influenced by extracellular matrix composition, cell junctions, tissue stiffness, and local signaling gradients [131]. Organoids therefore provide a means to study how the physical properties of tissues regulate viral infection rather than viewing viral entry and spread solely as receptor-mediated processes.

**Figure 4:**
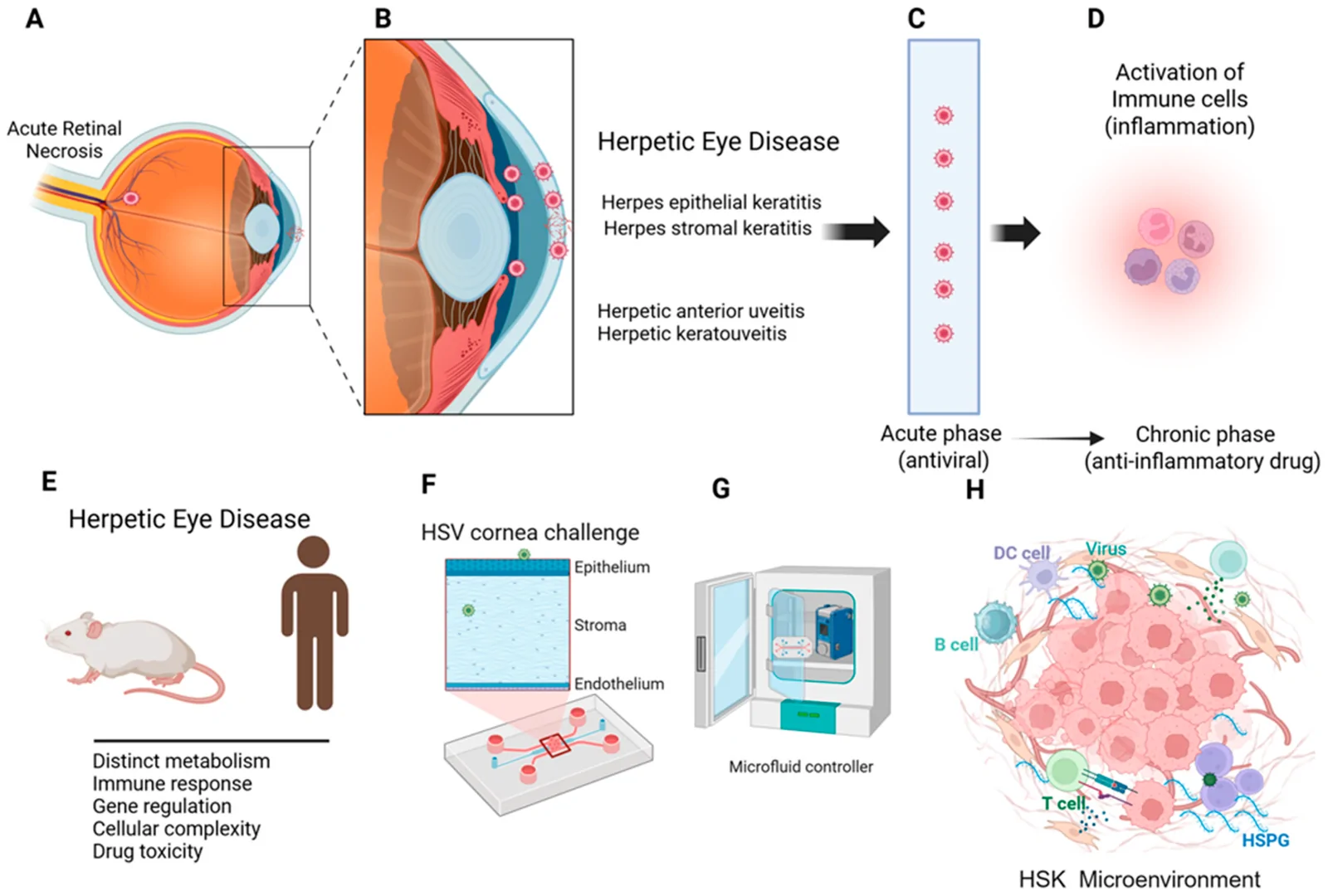
Significance of OOCT in modeling herpetic eye disease. (**A**,**B**). Herpes simplex virus (HSV) targets multiple ocular cell types and tissues, resulting in progressive vision loss and, in severe cases, blindness. (**C**,**D**). Herpetic eye disease progresses through a biphasic clinical disease with an acute phase characterized by active viral replication, followed by a chronic phase driven by dysregulated immune responses that compromise visual function. Accordingly, treatment strategies involve antivirals, anti-inflammatory corticosteroids, or combination regimens tailored to disease stage. (**E**). Limitations of traditional animal models, including significant differences between murine and human metabolism, immune responses, gene regulation, and drug toxicity, underscore the growing importance of OOCT as a physiologically relevant human-based platform. (**F**–**H**). Advances in 3D ocular organoid generation (corneal and retinal) and their integration into OOCT systems, especially with incorporation of immune components, enable detailed modeling of HSV-induced pathology, including herpetic stromal keratitis (HSK). These platforms support high-throughput drug screening and, when combined with glycan profiling approaches, provide a powerful framework for developing glycan-based therapeutics aimed at reducing inflammation and enhancing corneal wound healing and tissue repair. Created in BioRender. Tiwari, V. (2026) https://app.biorender.com/citation/698c962a0cce20c6222e3997.

The experimental flexibility of organoids also enables direct testing of host factors that control viral infection. Genetic approaches such as CRISPR-based gene editing and RNA interference, together with pharmacological inhibitors, can be used to determine how specific cytoskeletal proteins, motor proteins, receptors, or signaling pathways contribute to viral entry and intracellular transport. Because organoids contain different cell populations, these approaches can also reveal why some cell types are more susceptible to infection than others. Combining live-cell imaging with single-cell transcriptomics and proteomic analysis can further link viral behavior to cell-specific changes in gene expression and signaling, helping identify host pathways that may be targeted therapeutically. Organoid-on-chip systems extend these capabilities by adding controlled fluid flow, mechanical forces, and tissue interfaces that are difficult to reproduce in conventional organoids. Viral infection occurs within a physical environment in which shear stress, tissue deformation, barrier function, and biochemical gradients can influence receptor expression, viral entry, immune signaling, and dissemination [15]. By integrating human-derived organoids with microfluidic systems, these platforms allow infection to be studied dynamically and longitudinally. This is particularly relevant for viruses such as herpes simplex virus (HSV), which infects different tissues and can produce localized as well as disseminated disease [132]. Such systems may also provide new opportunities to investigate tissue-specific viral spread, persistence, and latency [94,133]. Ocular organoid and organ-on-chip models illustrate the value of this approach for studying tissue-specific viral disease (Figure 4). Human corneal organoids derived from embryonic or induced pluripotent stem cells can reproduce major corneal cell populations, including epithelial cells, stromal keratocytes, and endothelial cells, together with features of the native extracellular matrix [134]. These models provide a foundation for investigating how viral infection affects corneal barrier function, tissue organization, inflammation, and repair. More advanced “mini-cornea” models can reproduce transparent epithelial structures and corneal-specific differentiation programs [135]. When incorporated into microengineered cornea-on-chip platforms, these tissues can be exposed to controlled flow and other physical conditions, providing a more realistic environment for studying ocular drug delivery, HSV infection, and tissue repair [136].

The development of an ocular surface-on-chip containing both corneal and conjunctival tissues represents a further advance [137]. By incorporating a mechanically controlled blinking system, this platform reproduces important features of the ocular surface, including tear-film formation, mucin production, epithelial differentiation, and mechanical stimulation. Altering blinking frequency and humidity also reproduced inflammatory features associated with dry eye disease, including increased IL-1β, TNF-α, and MMP-9 [137]. Such systems demonstrate how organoid-on-chip technology can move beyond modeling viral replication alone to investigate the interaction between infection, tissue mechanics, inflammation, and repair. This is particularly important for HSV ocular disease, in which tissue damage and inflammation can persist even after viral replication is controlled [138]. Similar advances have been made in skin-on-chip models of HSV infection. These systems reproduce the stratified organization of human skin and allow infection and host responses to be examined under controlled conditions [139]. Studies have shown that basal keratinocytes are more susceptible to HSV infection than differentiated keratinocytes, consistent with differences in cellular organization and junction formation. Infection also induces IL-8 production and promotes neutrophil recruitment, demonstrating that these models can reproduce important aspects of the inflammatory response. Treatment with acyclovir reduces viral replication in a dose- and time-dependent manner, supporting the use of skin-on-chip systems for antiviral evaluation [139]. Incorporating patient-derived epithelial and immune cells could further enable individualized studies of viral susceptibility and therapeutic response. Neural organoids provide another important example of how three-dimensional tissue organization can reveal virus-specific mechanisms. Human brain organoids combined with single-cell analysis, electrophysiology, and imaging have shown that HSV-1 infection disrupts neuronal integrity, tissue organization, and cell-specific gene expression [96]. Although acyclovir effectively suppresses viral replication, it does not completely prevent neuronal and neuroepithelial injury. The ability of anti-inflammatory agents such as necrostatin-1 and bardoxolone methyl to reduce tissue damage suggests that controlling host inflammatory pathways alongside viral replication may provide greater therapeutic benefits. HSV-1 infection of cerebral organoids has also been associated with amyloid-β accumulation, reactive glial responses, neuroinflammation, and neuronal loss, providing a human-relevant system for examining links between viral infection and neurodegenerative processes [96]. Brain organoids have also provided important insights into virus-induced developmental injury. Infection with Zika virus (ZIKV) and HSV-1 can impair organoid growth through cell death and virus-specific changes in cellular programs [90,93]. ZIKV preferentially affects neural progenitor populations and produces developmental abnormalities, whereas HSV-1 can disrupt neuroepithelial organization and neuronal development. Interestingly, specific interferon treatments can partially restore organoid growth despite limited activation of classical interferon responses during infection. These findings demonstrate the ability of organoid models to distinguish virus-specific mechanisms of neural injury and to identify host responses that may be therapeutically manipulated.

Overall, organoid and organoid-on-chip systems provide a framework for connecting viral behavior with tissue architecture, cellular mechanics, host signaling, and tissue injury. Their major advantage is not simply that they reproduce more features of human tissues, but that these features can be experimentally controlled and measured over time. As immune cells, vascular components, patient-derived tissues, biosensors, and advanced imaging are incorporated, these platforms have the potential to define why viruses behave differently across tissues and individuals. This makes them particularly valuable for developing therapies that address not only viral replication but also the tissue-specific inflammation and damage that ultimately determines disease outcome.

## 7. Gut–Brain Axis as a Modulator of HSV-1 Neuropathogenesis: Mechanisms, Models, and Translational Implications

The gut–brain axis is a bidirectional communication network that transmits intestinal luminal information to the central nervous system (CNS) and efferent input from the brain through neural, endocrine, immune, and metabolic pathways. The vagus nerve (Xth cranial nerve) is known to function as the cargo that facilitates this bidirectional neurotransmission, with well-established roles in hunger and appetite regulation, metabolic handling of fuel substrates, and bioenergetics. Mechanistic approaches to study this axis have been met with challenges posed by the absence of models with the ability to recapitulate multiple organs, the complex neurocircuitry, and concurrent signaling pathways. Interactions between the gut microbiota and the host immune system are increasingly recognized as key determinants of susceptibility to viral diseases and neurological outcomes. Advances in sequencing and metabolomics have established this axis as a mechanistic framework through which gut microbiota regulate immune homeostasis, microglial maturation, blood–brain barrier (BBB) integrity, and systemic inflammation. While dysregulation of this axis has long been implicated in neurodevelopmental and neurodegenerative disorders, it has more recently emerged as a critical modulator of virus-induced neuropathology, with herpes simplex virus type 1 (HSV-1) serving as a particularly informative model.

HSV-1 is a highly prevalent neurotropic virus that establishes lifelong infection and causes a wide spectrum of neurological outcomes, ranging from asymptomatic latency to herpes simplex encephalitis (HSE), a severe inflammatory CNS disease associated with rapid neuronal injury, cognitive impairment, and substantial mortality despite antiviral therapy. Although HSV-1 molecular virology is well characterized, disease severity and long-term neurological sequelae are increasingly understood to depend on host-derived modulatory systems rather than viral replication alone. Innate immune responses, integrity of blood–brain barrier, and gut microbiota-derived metabolic signals collectively shape CNS vulnerability and inflammatory outcomes following infection.

Recent mechanistic studies have directly linked gut microbial metabolites to HSV-1-mediated neuroinflammation. Neurotropic HSV-1 infection induces mitochondrial dysfunction and suppresses PINK1–PRKN-dependent mitophagy, leading to accumulation of damaged mitochondria and microglial overactivation. This mitochondrial stress amplifies proinflammatory cytokine production and accelerates HSE progression. In contrast, nicotinamide *N*-oxide (NAMO), a gut microbial metabolite primarily produced by *Lactobacillus gasseri* and *Lactobacillus reuteri*, crosses the BBB and restores NAD^+^-dependent mitophagy in microglia. This mitochondrial quality control mechanism restrains microglia-mediated neuroinflammation, limits early HSV-1 infection in neuronal cells, and significantly attenuates disease severity. Consistent with this model, gut microbiota depletion exacerbates neuroinflammation, whereas exogenous NAMO administration suppresses HSE pathology. These findings identify gut microbiota-derived metabolites as intrinsic regulators of microglial homeostasis and HSV-1 neuropathogenesis.

Despite these advances, defining causal gut–brain mechanisms in HSV-1 infection have been constrained by limitations of traditional experimental models. Animal systems are confounded by species-specific microbiota and immune responses, while two-dimensional cultures fail to recapitulate tissue architecture and multicellular interactions. Human organoid technologies offer a powerful alternative. Brain organoids faithfully model HSV-1 neurotropism, neuroepithelial disruption, cytokine induction, and neuronal injury, while incorporation of microglia enables direct investigation of virus-induced microglial activation, inflammatory signaling, and mitochondrial quality control pathways such as mitophagy.

Intestinal organoids complement these models by recapitulating human gut epithelial barrier function, innate immune signaling, and metabolite transport. Although HSV-1 is not an enteric virus, gut inflammation and barrier dysfunction profoundly influence systemic immune tone and CNS susceptibility to neurotropic infection. Intestinal organoids therefore provide a human-relevant platform to examine how epithelial responses and metabolite flux shape downstream neuroinflammatory outcomes.

The full potential of organoid technology lies in integrated gut–brain and organ-on-a-chip systems that enable controlled coupling of intestinal and brain compartments. In the context of HSV-1, these platforms allow causal tracing of how gut-derived inflammatory mediators and microbial metabolites modulate BBB integrity, microglial activation, mitochondrial stress responses, neuronal survival, and potentially HSV-1 latency and reactivation. Such insights are not accessible through isolated organoid or animal models.

Beyond mechanistic discovery, gut–brain organoid systems offer substantial translational value. Antiviral agents, immunomodulators, probiotics, postbiotics, and mitochondrial-targeted therapies can be evaluated in human-specific platforms that capture the complexity of gut–brain–immune interactions and improve therapeutic predictability.

In summary, the gut–brain axis is a critical determinant of CNS outcomes in HSV-1 infection, with microbiota-derived metabolites such as NAMO shaping microglial neuroinflammatory responses through mitochondrial quality control pathways. Integrated gut–brain organoid platforms provide an unprecedented opportunity to causally dissect these interactions, bridging microbial ecology, immunometabolism, and neural biology, and accelerating the development of targeted therapies for HSV-1-associated neurological disease.

## 8. Multi-Organ-on-a-Chip Systems: Advancing Preclinical Drug Evaluation and Translational Toxicology

**Figure 5:**
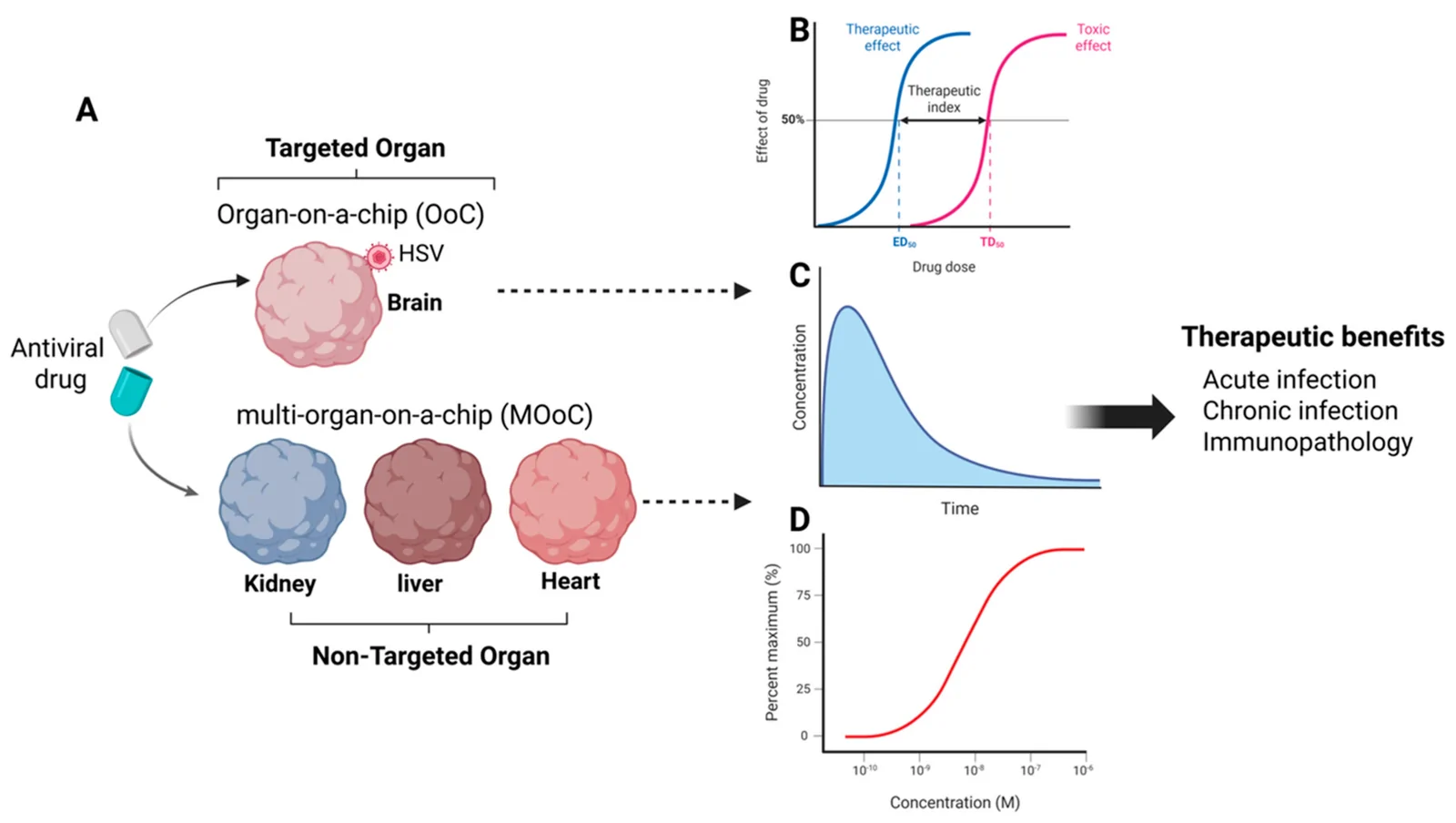
Comparative evaluation of antiviral drug responses using organ-on-chip (OoC) and multi-organ-on-a-chip (MOoC) platforms. (**A**). Schematic representation of the experimental workflow highlighting the sequential use of HSV-1-targeted organs (OoC) and non-targeted organs (MOoC systems) in antiviral drug development. Initial screening is performed in single-organ OoC models to assess antiviral efficacy in a target tissue (e.g., brain organoid for neurotropic viruses such as HSV), enabling high-resolution analysis of viral replication dynamics and drug potency in a tissue-specific context. While OoC platforms provide critical insights into localized drug efficacy and cellular responses, they lack systemic integration required to capture whole-body drug distribution. MOoC-based modeling involving effective dosage (**B**) including pharmacokinetic–pharmacodynamic (PK–PD) studies (**C**,**D**) would be ideal to identify therapeutic dosages. By integrating multiple organoids (e.g., brain, liver, kidney, and cardiac tissues) within a connected microfluidic network, MOoC systems enable the assessment of inter-organ crosstalk, drug biotransformation (particularly hepatic metabolism), and secondary toxicities in non-target tissues. This approach is particularly critical for antiviral drug evaluation, where compounds demonstrating efficacy in target tissues may induce off-target effects upon systemic exposure. For example, in the context of HSV infection, antiviral candidates optimized for brain organoid efficacy must be further evaluated in MOoC platforms to determine their impact on hepatic metabolism, renal clearance, and cardiotoxicity, especially under chronic exposure and variable dosing regimens [96]. Collectively, OoC platforms are ideally suited for target-specific efficacy screening and mechanistic studies, whereas MOoC systems provide a systems-level framework for integrated pharmacokinetic, pharmacodynamic, and toxicological evaluation, thereby enhancing translational predictability and reducing the risk of late-stage clinical failure. Created in BioRender. Tiwari, V. (2026) https://app.biorender.com/citation/6a73b3a709c3c565846510f9.

Multi-organ-on-a-chip (MOoC) systems extend organoid and organ-on-chip technologies from tissue-specific models toward integrated studies of human physiology, drug responses, and systemic toxicity [140]. By connecting human-derived organoids or tissue modules through controlled microfluidic networks, these platforms reproduce selected features of organ–organ communication, circulation, metabolism, and tissue-specific responses that are difficult to capture in conventional cell cultures. This interconnected architecture enables simultaneous assessment of antiviral efficacy, drug metabolism, pharmacokinetics, pharmacodynamics, and toxicity across multiple tissues within a single experimental system (Figure 5). Thus, while individual organ-on-chip models provide detailed information about infection and therapeutic responses within a target tissue, multi-organ microfluidic systems add the critical dimension of inter-organ interactions.

This capability is particularly important in antiviral drug development, where therapeutic activity must be considered together with drug metabolism, distribution, and potential toxicity in non-target organs. In a multi-organ platform, a compound can undergo hepatic metabolism before reaching downstream tissues, allowing investigators to determine how biotransformation affects antiviral activity and whether metabolites produce adverse effects elsewhere [140]. Such models can therefore provide information that cannot be obtained from a single-cell or single-organ assay, including whether a candidate drug maintains therapeutic activity after metabolism and whether effective antiviral concentrations can be achieved without damaging other tissues. The need for improved human-relevant toxicity models is substantial. Drug-induced liver injury is a major cause of drug withdrawal and clinical failure [141]. Although animal models are widely used for safety assessment, species-specific differences in drug metabolism and hepatic responses limit their ability to predict human hepatotoxicity, with some studies estimating that only approximately 40–50% of clinical hepatotoxicity can be predicted using in vivo animal models [141]. Drug-induced kidney injury is another important cause of drug attrition and accounts for a substantial proportion of acute renal failure. A wide range of commonly used agents, including cisplatin, nonsteroidal anti-inflammatory drugs, antibiotics, and contrast agents, can damage the kidney. Similarly, drug-induced neurotoxicity can involve both structural neuronal injury and functional disturbances such as altered electrical activity or seizures, contributing significantly to discontinuation of drug candidates during development [142]. These limitations have encouraged the development of human-based hepatic, renal, and neuronal in vitro models, including those derived from human-induced pluripotent stem cells, to improve early prediction of organ-specific toxicity. MOoC platforms provide an opportunity to integrate these tissue-specific models into a connected physiological system. Rather than evaluating hepatic, renal, cardiac, or neural toxicity independently, interconnected tissues can be exposed to the same circulating compound and its metabolites. This allows investigators to follow how drug metabolism in one organ influences responses in another. Continuous perfusion can further reproduce prolonged exposure and enable real-time assessment of tissue integrity, metabolic activity, inflammatory signaling, barrier function, and cellular injury [140]. Importantly, this approach can distinguish direct toxicity from adverse effects that emerge only after drug metabolism or inter-organ signaling. The same principle can be applied to antiviral efficacy. Organ-on-chip systems can model viral replication, tissue injury, host responses, and therapeutic activity within the primary site of infection, while linked organs can reveal how systemic treatment affects other tissues. This is particularly relevant for viruses that produce localized and disseminated diseases or for antiviral agents that require hepatic activation or undergo extensive metabolism. Connecting respiratory, intestinal, hepatic, renal, neural, or reproductive tissue models could therefore provide a more complete picture of both viral disease and therapeutic response than isolated infection models. An additional advantage is the potential to incorporate patient-derived organoids into these interconnected systems. Organoids generated from individuals with different genetic backgrounds or disease characteristics could be used to examine differences in viral susceptibility, drug metabolism, therapeutic response, and toxicity. This creates an opportunity to move beyond population-level predictions toward patient-specific evaluation of treatment strategies. In this context, MOoC platforms could become particularly valuable for precision medicine by providing a controlled human system in which efficacy and safety are evaluated using tissues that retain patient-specific biological characteristics. The translational value of these platforms will ultimately depend on their ability to provide reproducible and decision-relevant information. Standardized tissue composition, flow conditions, drug dosing, exposure times, analytical endpoints, and performance criteria will be necessary to compare results across platforms and laboratories. Integration with real-time biosensors, high-content imaging, single-cell analysis, and computational modeling may further improve the ability to monitor drug responses and predict clinical outcomes. Importantly, the objective is not to reproduce every feature of the human body, but to develop well-characterized systems that reliably answer isolated, specific questions in drug discovery and development. Taken together, multi-organ-on-a-chip systems provide an important bridge between conventional in vitro assays and the systemic complexity of human drug responses. By linking viral infection with drug exposure, metabolism, therapeutic efficacy, inter-organ signaling, and toxicity, these platforms can provide a more comprehensive assessment of candidate antivirals before clinical testing [140,141,142]. Their continued development could reduce late-stage drug failure, improve identification of effective therapeutic combinations, and accelerate the selection of safer antiviral candidates. More importantly, integration of patient-derived tissues may establish MOoC systems as a foundation for precision antiviral medicine, in which therapeutic decisions are informed by human-specific and patient-specific responses rather than relying predominantly on animal models or simplified cell cultures.

## 9. Building a National Human Organoid Infrastructure and Research Community

The next generation of drug discovery will require human-based in vitro systems that better capture the complexity and diversity of human biology. The NIH’s Standardized Organoid Modeling (SOM) initiative represents an important step toward making organoid models more reproducible, scalable, standardized, and accessible. Building on this foundation, a future National Human Organoid Biobank Initiative (NHOBI) could establish a coordinated resource of well-characterized, cryopreserved organoids derived from healthy and disease-affected individuals representing diverse genetic, demographic, and clinical backgrounds. With appropriate consent and privacy protections, these organoids could be linked to genetic, molecular, clinical, and treatment-response information, creating a dynamic resource for studying disease mechanisms, host–pathogen interactions, drug efficacy, toxicity, and patient-to-patient variation. Importantly, broad access to validated organoid models would allow investigators, including those at resource-limited institutions, to conduct human-centered research without the substantial time and cost required to establish these systems independently. Such a resource could also strengthen preparedness for emerging infectious diseases by enabling rapid investigation of pathogen tropism, tissue injury, host responses, and therapeutic interventions.

SOM and NHOBI could therefore serve complementary functions: SOM could establish standards for generating and validating organoids, while NHOBI could preserve and provide access to the diversity of human biological models needed for meaningful research. However, infrastructure alone will not be sufficient. A strong scientific community and workforce will be essential to develop, share, and translate these technologies. Ultimately, the future of human-centered biomedical research will depend on both representative biological resources and the scientific community capable of using them. Together, standardized organoid frameworks such as SOM, a future national biobank such as NHOBI, and a connected in vitro biology community could provide a durable foundation for more predictive disease modeling, safer drug development, and improved responses to emerging health challenges.

## 10. Challenges, Standardization, and Future Directions of Organoid-on-Chip Systems in Virology

Organoid-on-chip (OOCT) systems represent an important step toward developing more human-relevant models of viral disease. By combining human organoids with microfluidic technology, these platforms can reproduce tissue architecture, cell polarity, barrier function, controlled microfluidic flow, mechanical forces, extracellular matrix (ECM) interactions, and communication between different cell types. These features are particularly relevant to virology because viral infection and disease progression depend not only on viral receptors and intracellular signaling but also on the surrounding tissue environment. OOCT systems therefore provide a useful platform for studying viral entry, dissemination, tissue injury, and host responses under conditions that more closely resemble human tissues than conventional two-dimensional cultures or static organoids [15,118]. However, the increased complexity of OOCT systems also presents several challenges. Organoids generated from different donors or batches can vary in cellular composition, maturity, and function, which may affect viral susceptibility and treatment responses [143]. Many organoids also resemble developing tissues rather than fully mature adult organs, limiting their ability to model chronic, age-related, or tissue-specific viral disease. Establishing clear criteria for organoid identity, maturity, function, and viral susceptibility will therefore be important for improving reproducibility. The microfluidic component introduces additional variability through differences in chip design, membrane properties, ECM composition, flow rates, and culture conditions. Specialized equipment, technical expertise, and biosafety requirements can further increase cost and limit accessibility [144,145]. Immune integration is another challenge. Adding macrophages, microglia, circulating immune cells, or other immune components can improve the study of inflammation and host responses, but these components should be included based on the specific research question rather than simply increasing model complexity. Scalability also remains a limitation, as many OOCT systems are technically demanding and support fewer experimental conditions than conventional multiwell cultures. Standardized organoid production, modular chip designs, automated fluid handling, and higher-throughput formats could help address these limitations.

Standardization and validation will be essential for moving OOCT systems from specialized laboratories into broader biomedical and pharmaceutical applications. Rather than developing a single universal platform, the field should establish common criteria for organoid identity and maturity, cellular composition, barrier function, microfluidic conditions, viral inoculation, assay endpoints, and reproducibility. Detailed reporting of chip design, ECM composition, culture conditions, and mechanical parameters will also improve reproducibility across laboratories. Regulatory developments, including the FDA Modernization Act 2.0, have created new opportunities for human-relevant models and New Approach Methodologies (NAMs). However, biological complexity alone is not sufficient to establish an OOCT system as a useful NAM. Each model should have a clearly defined context of use and demonstrate reproducible and predictive performance. Validation should therefore be fit-for-purpose and focused on specific applications, such as antiviral efficacy, tissue toxicity, mechanisms of viral injury, or therapeutic responses. Future OOCT platforms will increasingly incorporate vascular, immune, and interconnected organ systems to study viral dissemination, drug exposure, immune-cell trafficking, and multi-organ disease. Patient-derived organoids may also help identify differences in viral susceptibility and therapeutic responses among individuals. Integration with single-cell RNA sequencing, spatial transcriptomics, proteomics, and glycomics can provide greater insight into how viral infection alters individual cell populations, tissue organization, glycocalyx composition, and immune signaling. Importantly, OOCT models can reveal mechanisms that may not be detected in conventional cell cultures. For example, vascularized intestinal chips have identified STEAP3 as a factor involved in viral entry, while respiratory OOCT models have shown that mechanical stretching can influence viral infection and host responses [146,147]. These findings demonstrate that tissue environment can be an important determinant of viral infection and disease.

The applications of OOCT technology also extend beyond antiviral drug discovery. Human tonsil and spleen organoids have been used to evaluate vaccine antigens and adjuvants and to study how pre-existing immune memory influences vaccine responses [148,149]. Similarly, multi-organ systems linking liver, kidney, and target tissues can provide information about drug metabolism, distribution, antiviral activity, and toxicity that is difficult to obtain using conventional models [150]. These approaches could help identify ineffective or toxic candidates earlier in drug development. Ultimately, the value of OOCT technology will depend not on how closely these systems reproduce human tissues, but on whether they improve the prediction of therapeutic efficacy, toxicity, and patient outcomes. Integration of patient-derived organoids, immune and vascular components, multi-omics, automation, and artificial intelligence could make OOCT platforms more reproducible and scalable [151]. As the field advances, population-representative organoid biobanks could further support human-relevant disease modeling and drug discovery. OOCT systems should therefore complement, rather than replace, conventional cell cultures and organoids. Their greatest value will be in research questions where tissue architecture, microfluidic flow, mechanical forces, vascular interactions, barrier function, immune responses, or communication between organs are important. With appropriate standardization and validation, OOCT systems could help bridge the gap between molecular virology and human disease and support more predictive, patient-centered antiviral development.

## Data Availability

No new data were created or analyzed in this study.
